# Evaluation of anatomic landmarks to increase precision performing a mini-hemilaminectomy—an *ex vivo* study in dogs

**DOI:** 10.3389/fvets.2024.1385249

**Published:** 2024-05-13

**Authors:** Stefanie Brechbühl, Benjamin Husi, Sebastian Knell

**Affiliations:** ^1^Tierklinik Aarau West AG, Oberentfelden, Switzerland; ^2^Clinic for Small Animal Surgery, Vetsuisse Faculty of Zurich, Zurich, Switzerland

**Keywords:** mini-hemilaminectomy, intervertebral disk, accessory process, dog, thoracolumbar spine

## Abstract

The mini-hemilaminectomy is a frequently used surgical technique for decompressive disk surgery on dogs. The aim of the study was to assess landmarks in the canine thoracolumbar spine to perform a mini-hemilaminectomy, with the aim of achieving optimal exposure of the ventral aspect of the vertebral canal. We hypothesized that the accessory process is a useful landmark for the identification of the level of the vertebral canal floor (VCF) and for decreasing surgical time. To define the level of the VCF, different landmarks and their distance to the VCF from computed tomography images of 40 mature chondrodystrophic dogs were evaluated in the first part of the study. To test the predefined landmarks, a cadaveric experiment was subsequently performed in the second part of the study. An experienced surgeon and a second-year surgical resident performed mini-hemilaminectomies as precisely as possible, with and without using the landmark values. Surgery time, precision of the mini-hemilaminectomy, and iatrogenic damage of the spinal nerve roots were compared between the two groups. Based on the results in the first part of the study, the distance from the dorsal border of the accessory process to the VCF (DBAP-VCF) was chosen as a landmark due to the good intra- (0.96) and interobserver (0.83) agreement. However, the distance is highly variable between breeds. In the second part of the study, using the DBAP-VCF landmark value did not influence the surgery time in both surgeons (*p* = 0.467, *p* > 0.99). An improved accuracy of the VCF was seen for the surgical resident with limited experience (*p* = 0.014), but not for the experienced surgeon (*p* = 0.926). For both surgeons, the spinal nerve roots were injured in 20% of the cases unrelated to the use of landmark values. In conclusion, this study suggests that the DBAP-VCF has been described as a breed-specific landmark that can be determined in CT with good agreement. Using the previously evaluated landmark values can help improve precision in decompressive spinal surgery for a surgeon with limited experience without prolonging surgical time.

## 1 Introduction

Intervertebral disk disease is a common condition in dogs, with an overall incidence ranging from 2.3 to 2.7% (1, 2). Breeds with chondrodystrophy, such as Dachshund, Beagle, Pekingese, Jack Russell Terrier, and French Bulldog, are most susceptible (3, 4). The pathology typically involves chondroid degeneration of the nucleus pulposus and rupture of the annulus fibrosus, resulting in herniation of nucleus material into the vertebral canal and subsequent spinal cord compression, primarily in the caudal thoracolumbar region between the twelfth thoracal vertebra (T12) and third lumbar vertebra (L3) (5). In 70–90% of the cases, the material is localized on the ventral, or ventrolateral, aspect of the vertebral canal (6, 7).

The preferred treatment for patients with severe, ongoing, or progressive neurologic deficits is surgical decompression (5). Various techniques for decompressing the spinal cord in the thoracolumbar area exist, with hemilaminectomy and mini-hemilaminectomy being the most commonly employed (6). Some studies suggest that the mini-hemilaminectomy more effectively reduces spinal cord compression and leaves less residual disk material in the spinal canal compared to the traditional hemilaminectomy, although it is less popular than the hemilaminectomy among veterinary surgeons (8–10). The infrequent use of the seemingly more effective mini-hemilaminectomy may be attributed to its complexity, as the smaller bone window provides less visualization of the surgical target compared to the hemilaminectomy and demands greater precision from the surgeon to adequately access the vertebral canal (9). To date, no specific guidelines have been established to assist surgeons in determining the level of the vertebral canal floor (VCF) and positioning the bone window for optimal access to the spinal canal.

The purpose of this study is to define landmarks such as the accessory process in the thoracolumbar spine to optimize access to the ventral aspect of the vertebral canal. The first hypothesis is that using the accessory process as a landmark leads to a more precisely performed mini-hemilaminectomy relative to the VCF, as compared to cases where the accessory process is not used as a landmark. The second hypothesis suggests that utilizing the accessory process as a landmark will result in decreased surgical time.

## 2 Materials and methods

The study consisted of two parts:

- Part I: Computed tomographic (CT) measurements to define surgical landmarks for the optimal position of the mini-hemilaminectomy.
- Part II: a cadaveric study in which the surgical procedure was performed with and without previously defined and calculated landmark values.

### 2.1 Part I: CT measurements and definition of surgical landmark

#### 2.1.1 Study population

Multislice CT scans (SOMATOM Emotion 16-slice configuration, Siemens AG Medical Solutions, Erlangen, Germany) of the spinal cord from T11 until L4 in 40 skeletally mature chondrodystrophic dogs were included in the study. These patients had CT scans of the spine from T10 to the sacrum for reasons unrelated to the study and were analyzed using a DICOM viewer [Horos v3.3.6 (Horos Project)]. Breed distribution included 10 French Bulldogs, 10 Beagles, and a mixed population of 20 chondrodystrophic dogs. Exclusion criteria were surplus or block vertebras (11). Vertebral malformation, as frequently seen in French Bulldogs, was not an exclusion criterion *per se*, but the malformed vertebrae were excluded.

#### 2.1.2 Measurements

Based on CT measurements from T11 to L4 on these 40 dogs, landmark values were defined to precisely position the bone window for the mini-hemilaminectomy. Landmarks were chosen based on easy identification during surgery and a consistent relationship to the vertebral canal. To validate these landmarks, intra- and interobserver agreement was tested, and if the data confirmed repeatable precision for a particular measurement, then that landmark value was tested in the second cadaveric part of the study.

**Figure 1:**
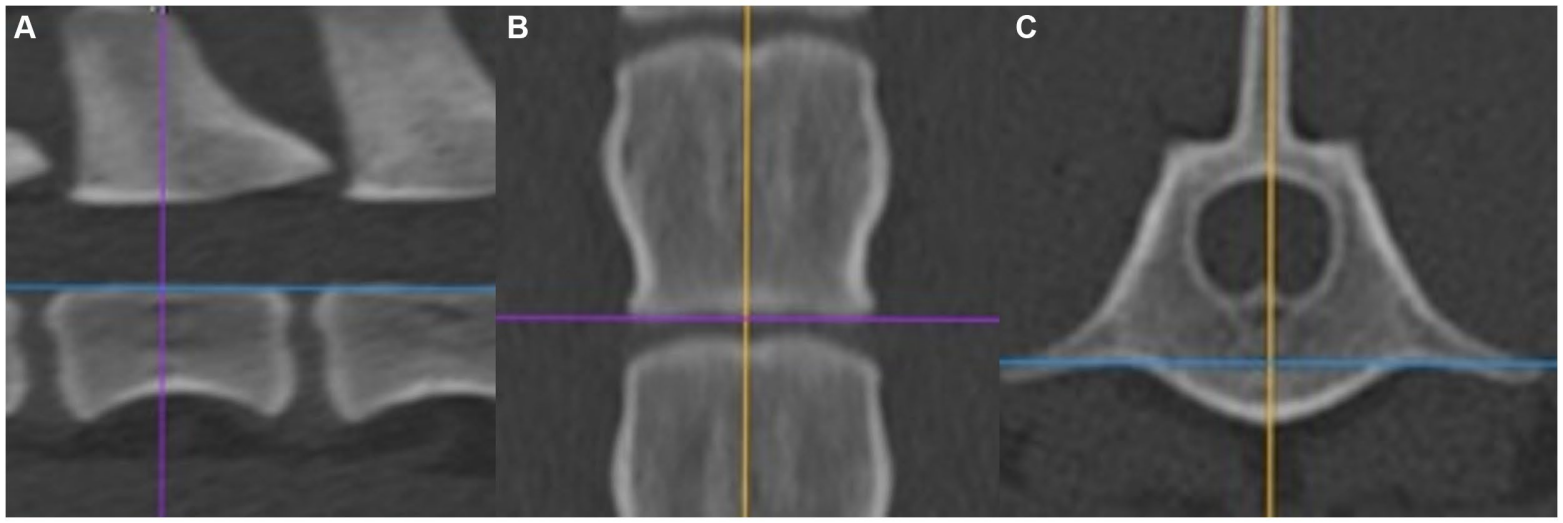
**(A)** Alignment in the sagittal plane. **(B)** Alignment in the dorsal plane. **(C)** Alignment in the transversal plane.

**Figure 2:**
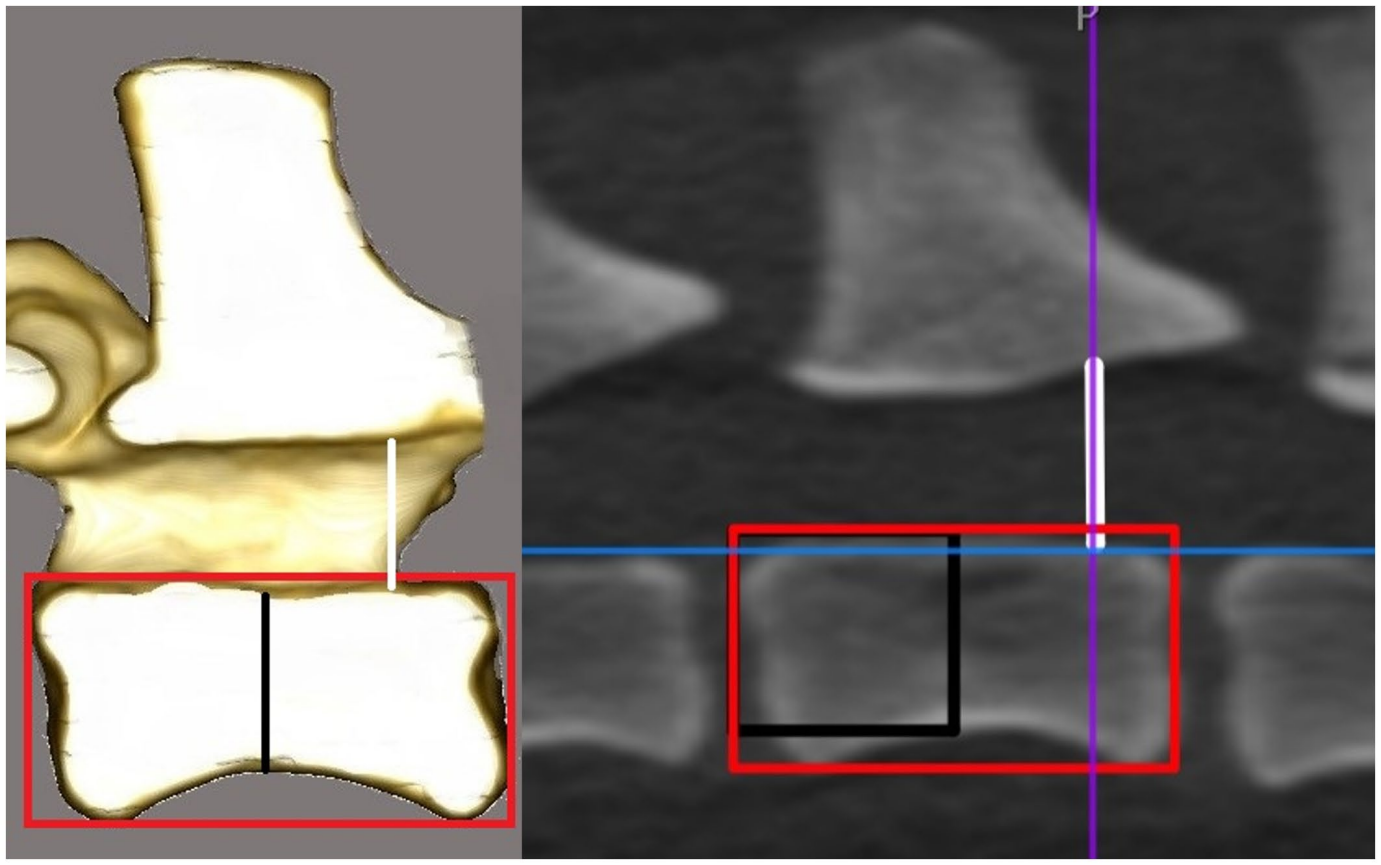
Drawing and CT image in the sagittal plane. The horizontal line of the red rectangle represents the vertebral body length. The black line in the drawing and the vertical line of the black rectangle in the CT image represent the vertebral body height. The white line represents the vertebral canal height at the cranial end of the vertebral foramen.

**Figure 3:**
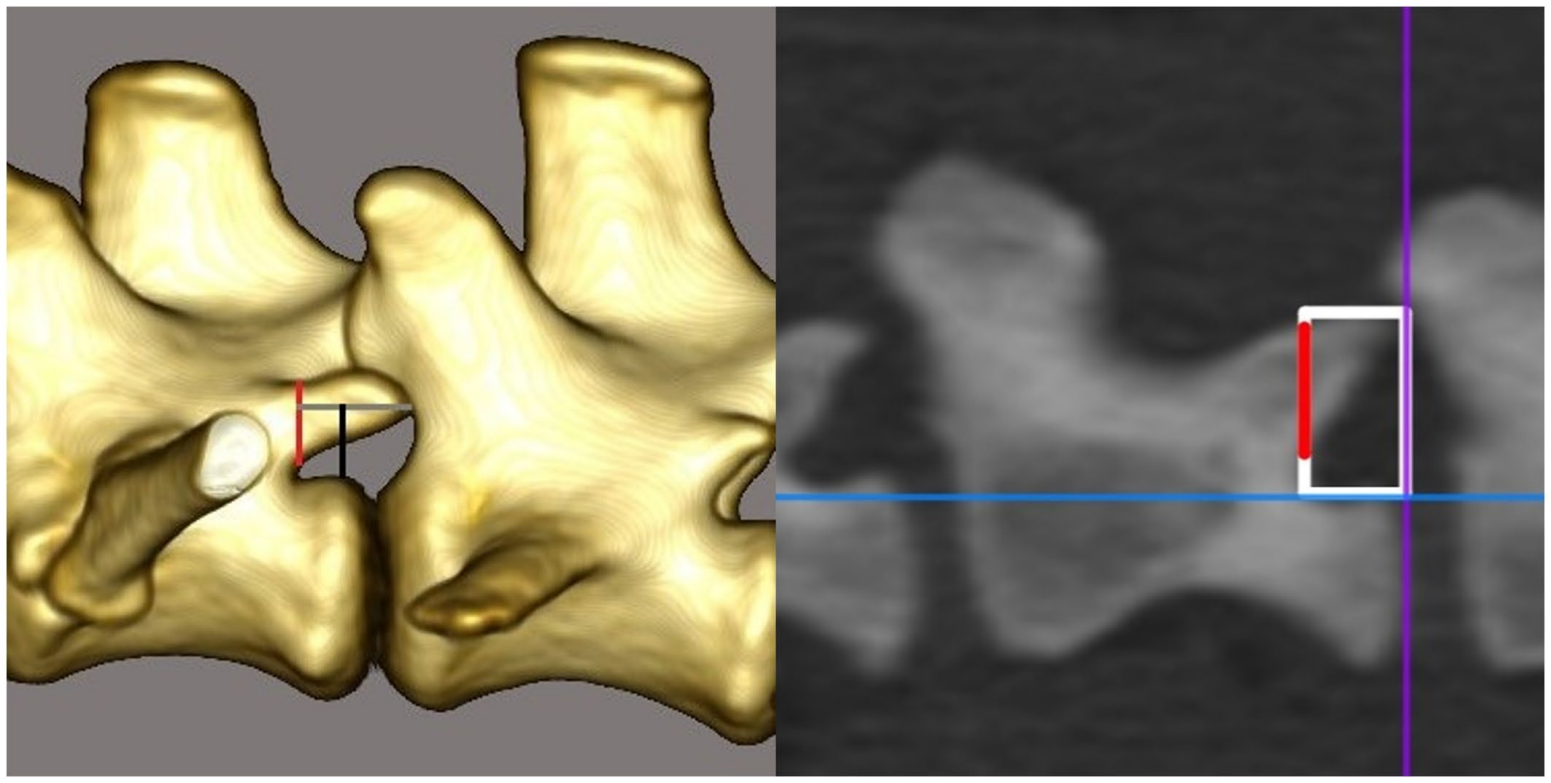
Drawing and CT image in the sagittal plane. The red line represents the accessory process height. The gray line in the drawing and the horizontal line of the white rectangle in the CT image, respectively, represent the accessory process length. The black line in the drawing and the vertical line of the white rectangle in the CT image, respectively, represent the distance between the tip of the accessory process and the vertebral canal floor. In the drawing, the accessory process extends over the intervertebral disk and therefore the measurement is taken in the region of the endplate of the corresponding vertebra.

**Figure 4:**
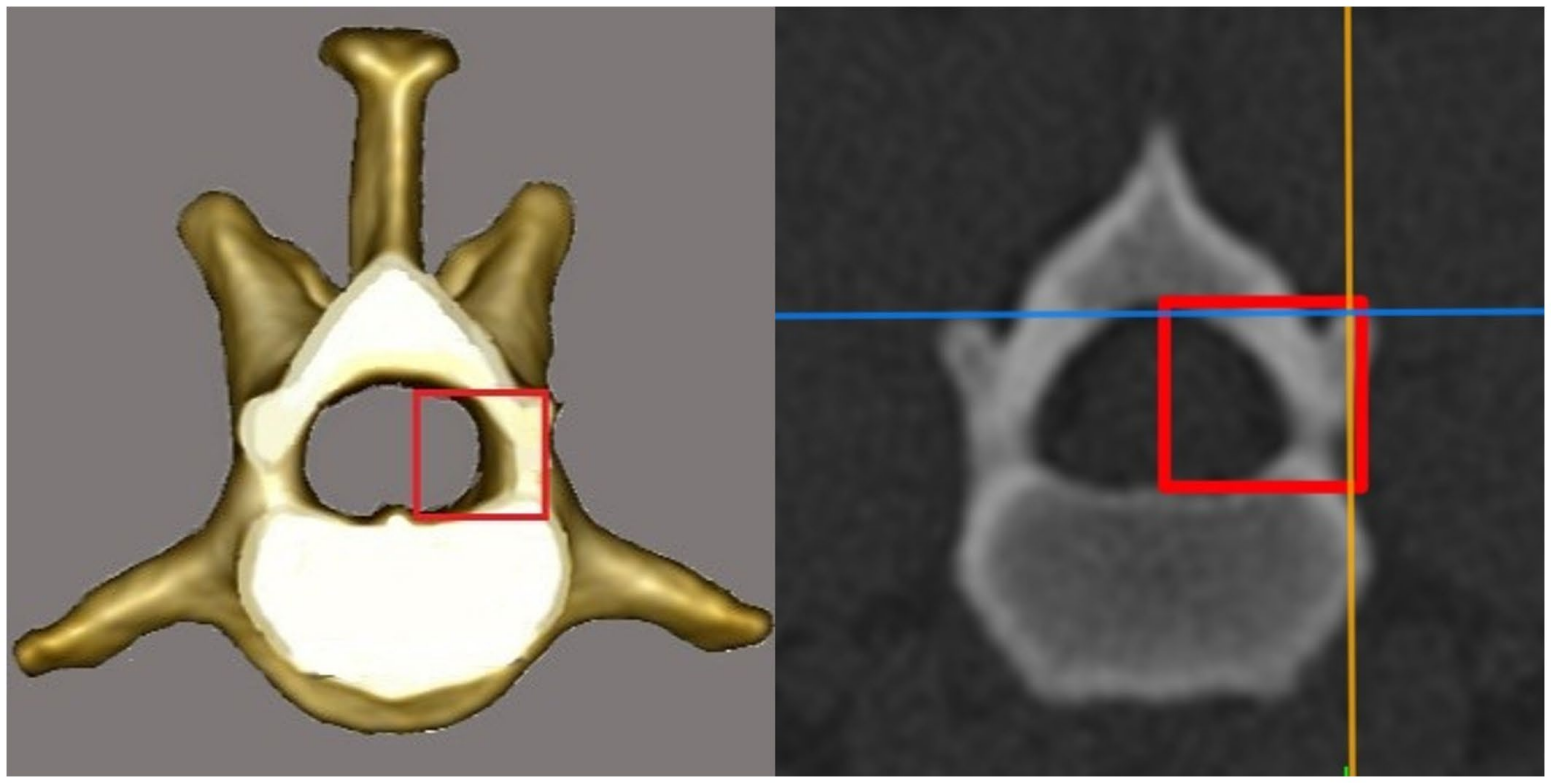
Drawing and CT image in the transversal plane. The vertical line of the red rectangle represents the distance between the dorsal border of the accessory process and the vertebral canal floor at the cranial end of the vertebral foramen.

The CT scans were evaluated by using the 3D multiplanar reconstruction mode in the DICOM viewer [Horos v3.3.6 (Horos Project)]. To achieve consistent measurements, images were aligned in a repeatable manner for each specimen and vertebra in the sagittal, dorsal, and transverse planes. We defined the blue reference line as the dorsal plane (dorsal reference line), the violet line as the transverse plane (transverse reference line), and the orange line as the sagittal plane (sagittal reference line). The dorsal reference line was aligned parallel to the floor of the vertebral canal in the sagittal plane (Figure 1A). The transverse reference line was aligned parallel to the caudal endplate in the dorsal plane (Figure 1B). In the transverse plane, the dorsal reference line was aligned immediately ventral to the origin of the transverse process with the sagittal reference line through the spinous process (Figure 1C). After determining the alignment of the reference lines, specific measurements for every vertebra were performed:

- the vertebral body length (VBL) is defined as the largest extent of the vertebral body in its median (Figure 2);
- the vertebral body height (VBH) is defined as the height of the vertebral body at 50% of the VBL in the median (Figure 2);
- the vertebral canal height (VCH) is defined as the height of the vertebral canal measured on the median of the vertebral canal at the cranial end of the vertebral foramen (Figure 2);
- the accessory process height (APH) is defined as the distance between the dorsal border of the accessory process (DBAP) and the cranial end of the vertebral foramen (Figure 3);
- the distance between the DBAP-VCF is defined as the distance in height between the DBAP at the cranial end of the vertebral foramen and the deepest point of the VCF (Figure 4);
- the accessory process length (APL) is defined as the sagittal extension of the accessory process, from the cranial end of the vertebral foramen to the tip of the accessory process (Figure 3);
- the distance between the tip of the accessory process and the VCF (TPA-VCF) is defined as the distance in height from the tip of the accessory process to the deepest point of the VCF. If the accessory process extended over the intervertebral disk, the vertical distance between the tip of the accessory process and the caudal end of the VCF of the corresponding vertebra was measured (Figure 3).

The measurements for the APH, APL, DBAP-VCF, and TPA-VCF were taken using the same methodology for the left and right sides. To evaluate the intra- and interobserver agreement, the left side of the vertebras of nine beagles were measured a second time by the author of the study for the intraobserver bias and by another author (SK) for the interobserver bias.

#### 2.1.3 Statistical analysis

Statistical analysis was performed using the Prism 9 Software (GraphPad Prism, San Diego, United States). For every value, the mean and the standard deviation were calculated. To evaluate the differences between the three groups and between the vertebras, a Bland–Altmann analysis was performed. Inter- and intraobserver agreement has been tested by using intraclass correlation coefficients. Intraclass correlation coefficient values from >0.8 were defined as good, in agreement with other publications (12–14).

### 2.2 Part II: cadaveric study

#### 2.2.1 Study population

Eight canine cadavers from chondrodystrophic dogs euthanized due to reasons unrelated to this study were used. They were stored at −18°C immediately after euthanasia and thawed at room temperature 36 h before the experiment. Specimens were chosen from breeds where measurements had already been performed in the first part of the study, and the mean results from these measurements were used.

#### 2.2.2 Experimental setup

The surgeries were performed by a second-year surgery resident with limited experience and a board-certified surgeon experienced in neurosurgery. No magnification devices were used during the surgeries. The eight cadavers were divided into four treatment groups (two each):

- Group 1: T11, T13, and L2 left side with landmark values and T12, L1, and L3 right side without landmark values.
- Group 2: T12, L1, and L3 left side without landmark values and T11, T13, and L2 right side with landmark values.
- Group 3: T11, T13, and L2 left side without landmark values and T12, L1, and L3 right side with landmark values.
- Group 4: T12, L1, and L3 left with landmark values and T11, T13, and L2 right side without landmark values.

The cadavers of each group were randomly assigned to the surgeon and procedure group. The first three mini-hemilaminectomies per cadaver were always performed without using the landmark values to avoid bias.

#### 2.2.3 Surgical technique

The animal cadaver was positioned in sternal recumbency with a tilt to one side (20°) with thoracic and pelvic limbs flexed and pulled slightly cranially. A skin incision was made 1.0–2.0 cm lateral to the dorsal midline, located where the articular processes are palpated through the skin and musculature. The length of the incision spanned T10 to L5. The incision was followed by performing a standard mini-hemilaminectomy approach to the vertebral column (15). The bone window was drilled with a pneumatic drill (Pen Drive, DePuy Synthes, 4436 Oberdorf, Switzerland) to access the inner cortex, and the vertebral canal was opened completely using Kerrison rongeurs. The goal was to create a mini-hemilaminectomy from the dorsal aspect of the accessory process to the VCF as precisely as possible without damaging the spinal nerve root. The cranial and caudal extent were approximately 40% of the vertebral body length. No previous CT measurements were performed to determine these dimensions.

**Figure 5:**
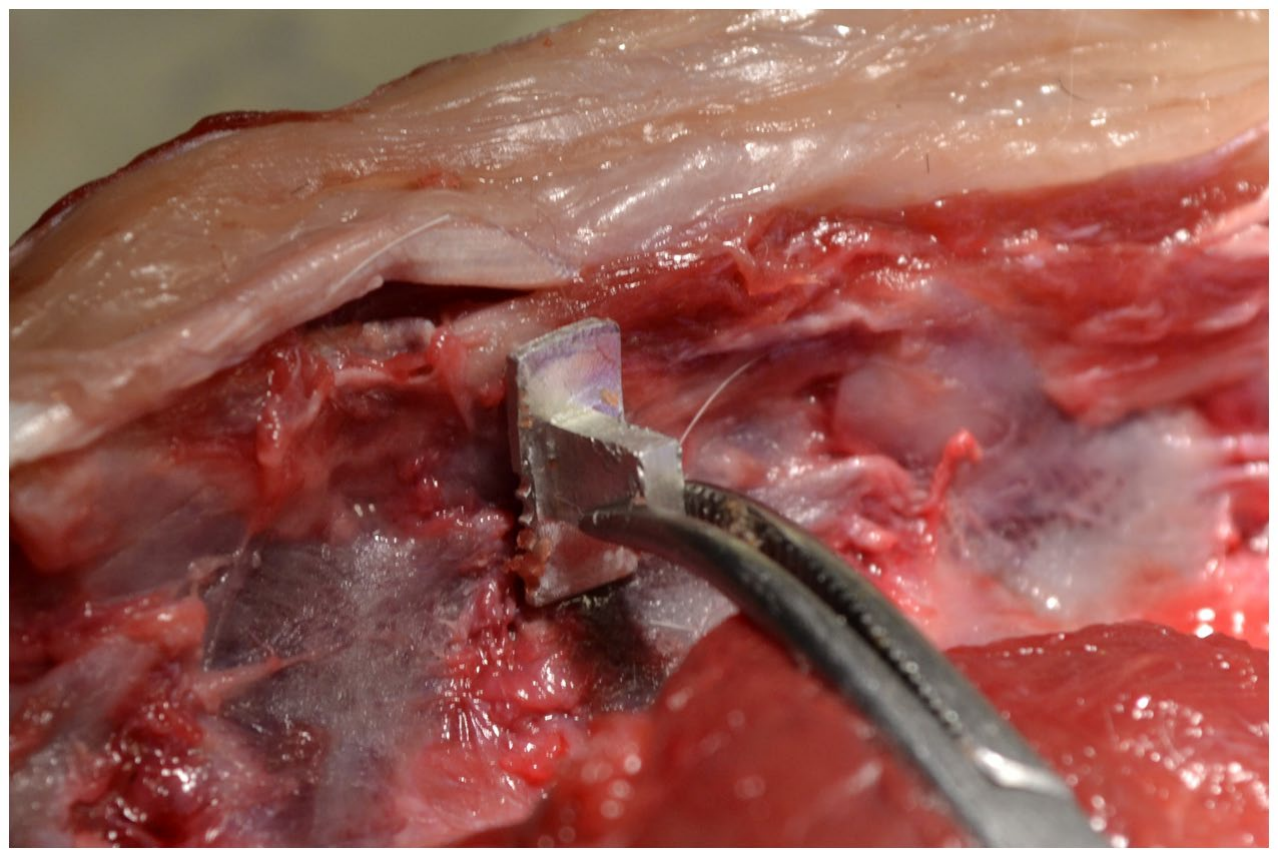
Use of the measuring device.

For the procedures using the landmark values, the surgeons received a measuring tool designed for this study and the calculated landmark value for each cadaver. The measuring tool is a 1-cm scale with a handle and marks at 5, 6, 7, 8, and 9 mm. It is constructed out of aluminum using a UCCNC molding cutter machine (Stepcraft D.420, STEPCRAFT GmbH &Co.KG, 58708 Menden, Germany). The surgeons received the landmark values, which were calculated from the measured values of Part I: for every breed and vertebra from Part I the mean DBAP-VCF was calculated and was then used as the landmark value for the specific vertebra and breed. If there was only one dog from the breed measured in Part I, the mean DBAP-VCF from the left and right side of the animal was used. The measuring tool was placed at the cranial end of the vertebral foramen (Figure 5). The expected VCF, determined by the landmark values, was marked with a needle in the remaining soft tissue and bony structures. The surgeons were asked to drill their bone window as prescribed and were not allowed to correct the position of the window after the first position was determined.

#### 2.2.4 Post-operative CT scans

Postoperative CT scans (SOMATOM Emotion 16-slice configuration, Siemens AG Medical Solutions, Erlangen, Germany) of the spine from T10 to L5 were performed in the sternal position. Scanning was performed at 120 kV and 250 mA, with a pitch of 0.688, rotation time of 0.75 s, and detector collimation of 16 × 0.75. Raw data were reconstructed using a bone algorithm with an increment of 0.5 mm. Images were exported to a workstation for data analysis with a DICOM viewer [Horos v3.3.6 (Horos Project)].

#### 2.2.5 Outcome measures

**Figure 6:**
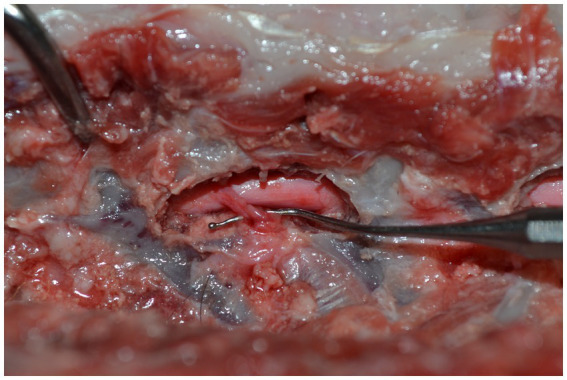
Intactness of the spinal nerve root is controlled after each surgery.

The total surgery time was defined as the time from the start of drilling until the bone window of the mini-hemilaminectomy was completed. The endpoint of the surgery was determined by the surgeon when the spinal cord was being inspected by palpation, and the surgeon was able to sufficiently palpate the ventral floor of the spinal canal at the assigned segment through the created window. After each surgery, the integrity of the spinal nerve root was confirmed (Figure 6).

For the procedures using the landmark values, the total time included picking up the measuring tool until the needle was placed to mark the ventral canal floor. This time was measured separately and included in the total surgery time. After each surgery, the integrity of the spinal nerve root was confirmed, and the surgeon was asked to judge the usefulness of the landmark values (not at all, little, or very).

**Figure 7:**
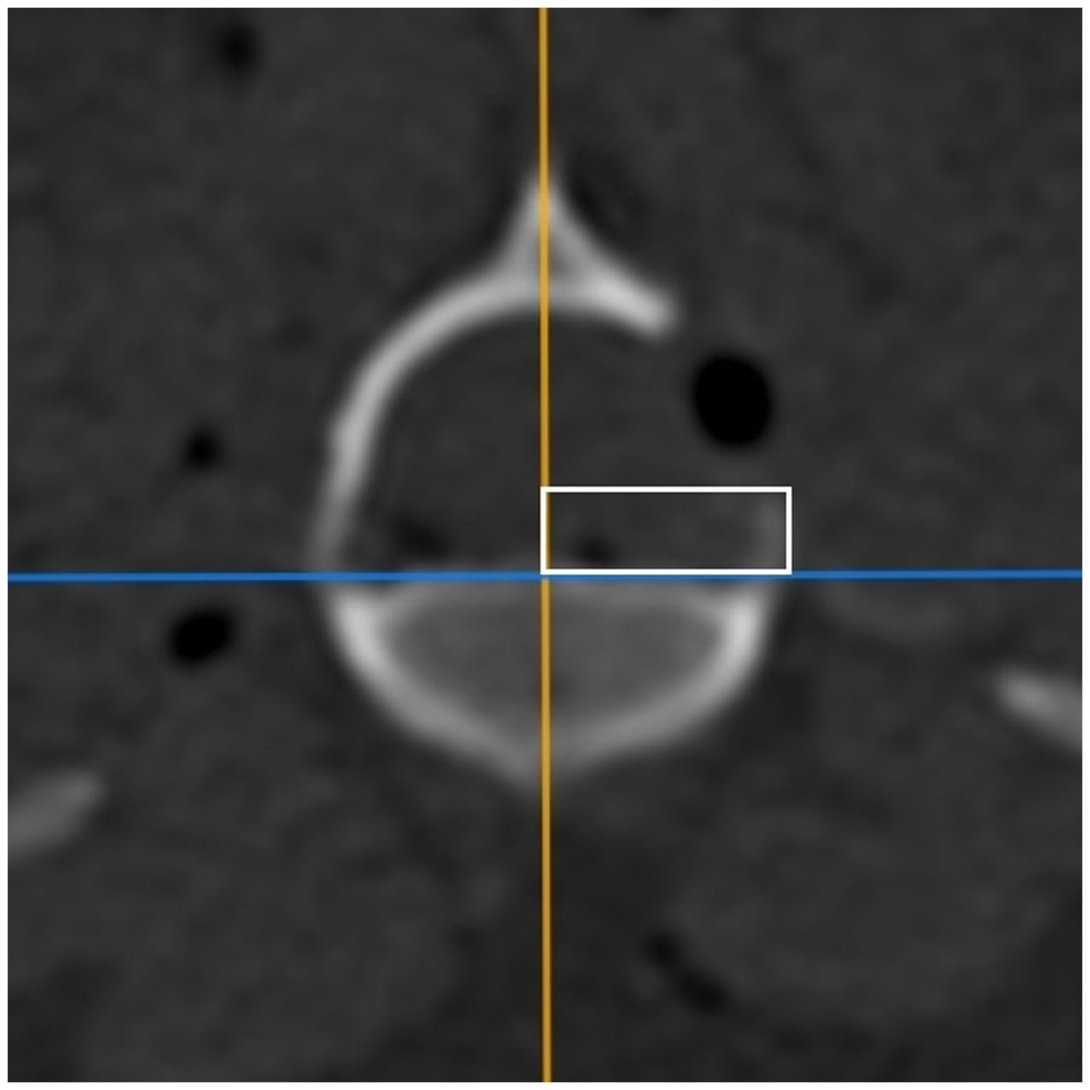
CT image in the transversal plane. The vertical line of the white rectangle represents the distance between the vertebral canal floor and the ventral border of the mini-hemilaminectomy at the level of the cranial end of the opposite foramen (ventral remnant).

In the postoperative scans, the ventral border of the mini-hemilaminectomy was evaluated by using the same image viewer program (Horos Project) as used in the first part of the study. The distance between the VCF and the ventral border of the mini-hemilaminectomy at the level of the cranial end of the opposite foramen was evaluated (ventral remnant) (Figure 7). Since the drilled window was not necessarily parallel to the vertebral floor, the mean value between the highest and lowest points was calculated.

#### 2.2.6 Statistical analysis

Statistical analysis was performed using the Prism 9 Software (GraphPad Prism, San Diego, United States). From the following values, the mean and the standard deviation were calculated:

- total time for the positioning of the measuring tool and marking of the ventral canal floor with a needle;
- total surgery time with and without given landmark values; and
- difference between the ventral border of the mini-hemilaminectomy and the VCF with and without given landmark values;

Due to the low number of specimens, it was assumed that the data were not normally distributed. A Wilcoxon signed-rank test was used to compare the different experienced surgeons regarding tool positioning, surgical time, and ventral remnant. To determine if there was a benefit by using the landmark values, surgical time for each surgeon was compared. A significance level of a *p* value of <0.05 was used.

## 3 Results

### 3.1 Part I: CT measurements and definition of surgical landmark values

#### 3.1.1 Patients

Of 40 dogs, 10 were Beagles, 10 were French Bulldogs, and the mixed chondrodystrophic group consisted of two Maltese dogs, four Bolonkas, three Havanese dogs, two Bichon Frise, two Cavalier King Charles Spaniels, two Dachshunds, two Chihuahuas, two Jack Russell Terrier, and one Shi Tsu.

#### 3.1.2 Excluded vertebrae

From the measurements of the French Bulldogs, two vertebrae from T11, four from T12, and one from T13 were excluded due to vertebral body malformations.

#### 3.1.3 Landmark measurements

The mean and standard deviation for all seven measurements of all groups and vertebrae are listed in Table 1. The intraobserver agreements were good for all measurements except the APH (0.57), but only the VBL, VBH, and DBAP-VCF values had a good interobserver agreement. Only VBL, VBH, and DBAP-VCF had an intra- and interobserver agreement from >0.8. VCH, APH, TPA-VCF, and APL have been excluded due to poor reproducibility.

**Table 1:** Mean and standard deviations of all measurements in mm.

Vertebra	Group	VBL		VBH		VCH		APH	
		Mean	SD	Mean	SD	Mean	SD	Mean	SD
T11	Mixed	12.785	2.06	6.044	1.263	5.495	0.576	2.013	0.506
	Beagles	16.683	0.666	8.418	0.539	7.422	0.347	1.908	0.376
	French bulldogs	14.934	0.758	8.616	0.965	8.293	0.492	3.768	0.446
T12	Mixed	13.415	2.085	6.149	1.24	5.552	0.756	2.855	0.727
	Beagles	17.489	0.55	8.286	0.608	7.55	0.387	2.806	0.941
	French bulldogs	15.33	0.82	8.297	0.754	8.157	0.349	4.055	0.597
T13	Mixed	14.136	2.164	6.02	1.33	5.642	0.729	3.277	0.760
	Beagles	18.671	0.819	8.26	0.53	7.513	0.431	3.818	0.537
	French bulldogs	16.436	0.921	8.081	1.159	8.352	0.516	4.772	0.781
L1	Mixed	15.069	2.432	5.981	1.32	5.905	0.723	3.328	0.749
	Beagles	20.219	0.794	8.218	0.576	7.579	0.576	4.262	0.351
	French bulldogs	18.204	1.041	7.687	1.083	8.809	0.655	4.422	0.454
L2	Mixed	15.73	2.579	6.367	1.409	5.872	0.889	3.264	0.784
	Beagles	20.993	0.852	8.663	0.575	7.847	0.573	4.469	0.360
	French bulldogs	19.329	0.988	8.16	1.06	8.956	0.75	4.638	0.474
L3	Mixed	16.068	2.628	6.495	1.541	6.434	0.705	3.275	0.888
	Beagles	21.814	0.944	8.61	0.53	8.392	0.708	4.425	0.327
	French bulldogs	20.344	1.159	8.434	1.166	9.187	0.792	4.380	0.716
L4	Mixed	16.554	2.767	6.445	1.681	6.591	0.988	3.093	0.818
	Beagles	22.332	0.807	8.682	1.02	8.139	0.658	4.238	0.275
	French bulldogs	20.387	1.253	9.53	1.598	9.33	0.617	4.464	0.751

Vertebra		DBAP-VCF		APL		TPA-VCF	
	Group	Mean	SD	Mean	SD	Mean	SD
T11	Mixed	5.84	1.352	2.711	0.73	5.633	1.349
	Beagles	7.865	0.651	1.94	0.54	7.438	0.606
	French bulldogs	7.903	0.731	3.871	0.823	7.759	0.677
T12	Mixed	5.718	1.565	4.806	1.016	6.085	1.639
	Beagles	7.405	0.733	4.175	1.275	7.704	0.726
	French bulldogs	7.841	0.965	6.1	0.705	7.683	0.886
T13	Mixed	5.195	1.105	5.91	1.351	5.672	1.202
	Beagles	7.244	0.786	5.534	0.849	7.922	0.657
	French bulldogs	7.482	0.957	6.982	0.85	7.673	0.923
L1	Mixed	5.214	1.062	4.856	1.929	5.642	1.169
	Beagles	7.001	0.533	5.081	0.932	7.68	0.353
	French bulldogs	7.007	0.684	5.793	1.727	7.442	0.65
L2	Mixed	5.038	0.969	4.372	1.732	5.611	1.109
	Beagles	6.701	0.625	5.24	1.071	7.399	0.551
	French bulldogs	7.038	0.681	5.58	1.821	7.537	0.77
L3	Mixed	5.018	0.822	3.153	1.358	5.198	0.86
	Beagles	6.385	0.484	4.149	1.354	6.92	0.492
	French bulldogs	6.815	0.685	3.985	1.406	7.349	0.744
L4	Mixed	5.179	0.775	2.798	0.904	5.236	0.898
	Beagles	6.488	0.478	4.526	1.238	7.052	0.564
	French bulldogs	6.776	0.743	3.646	1.205	7.107	0.737

The DBAP-VCF was chosen to be evaluated as a landmark in the experimental study because of its good intra- and interobserver agreement and its easiest intraoperative applicability.

**Figure 8:**
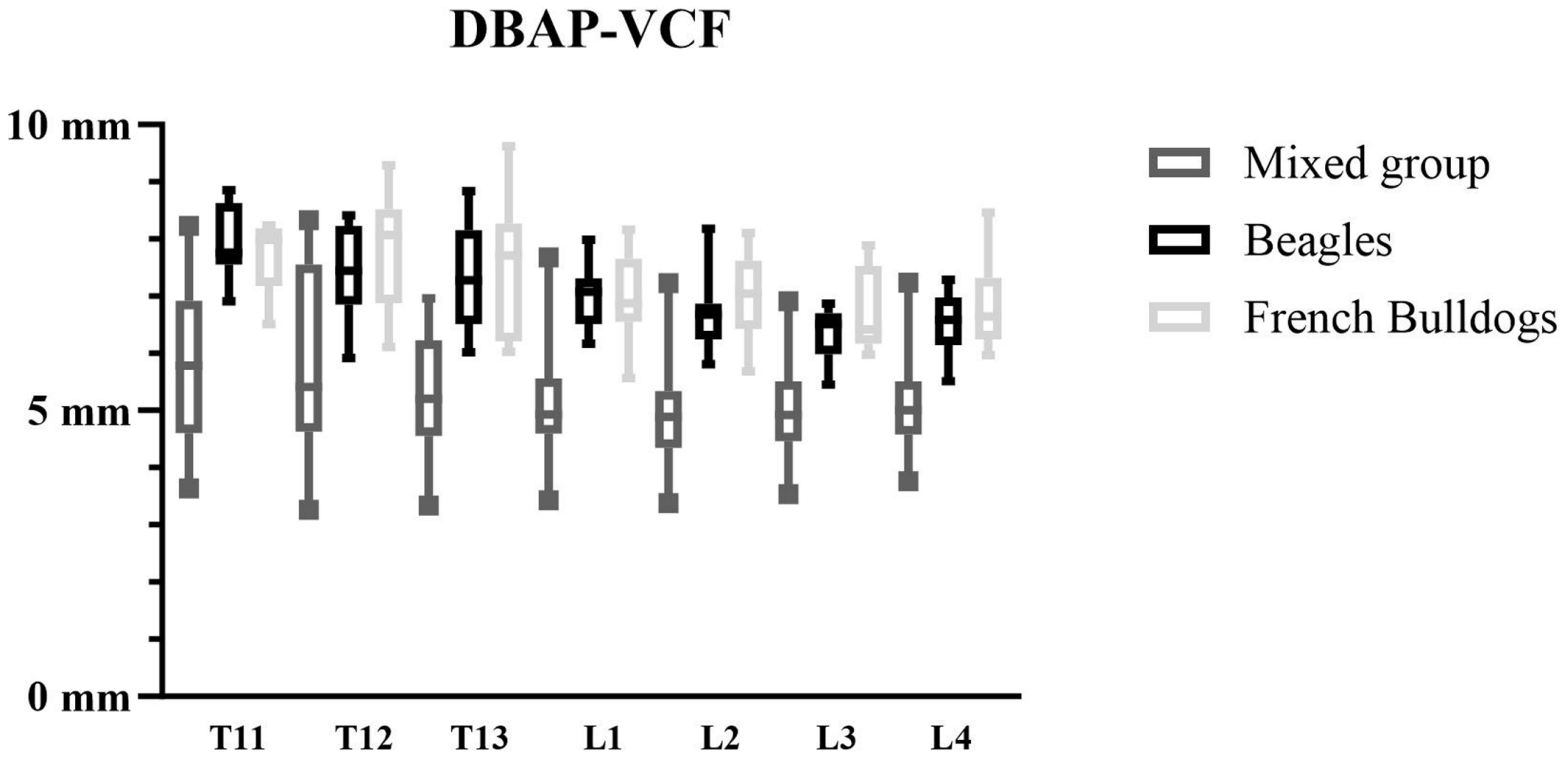
Boxplot shows that the DBAP-VCF value is breed-specific.

The DBAP-VCF was found to be larger in the thoracal region and decreased in size in the caudal direction until L4. The boxplot (Figure 8) illustrated that the mixed group has a much larger scattering of the DBAP-VCF value compared to the Beagle and the French Bulldog groups. For these reasons, a breed- and vertebra-specific DBAP-VCF value was the most adequate and used for the surgical part of the study as the landmark value.

### 3.2 Part II: Results of the cadaveric study

#### 3.2.1 Study population

The study population consisted of eight dogs: a Maltese dog, a Havanese dog, a Bichon Frise, a Cavalier King Charles Spaniel, a Dachshund, a Chihuahua, a Jack Russell Terrier, and a French Bulldog.

#### 3.2.2 Outcome measures

##### 3.2.2.1 Intraoperative measures

The total surgery time for the experienced surgeon was 250 ± 126 s without landmark values and 254 ± 79 s with landmark values and for the surgeon with limited experience, it was 408 ± 149 s without landmark values and 420 ± 117 s with landmark values (Table 2). There was no difference between the total surgery times with and without landmark values for both surgeons (*p* = 0.467 and *p* > 0.99).

**Table 2:** Mean measurements and standard deviations of the ventral remnant, total surgery time, and time of using the measuring tool.

	Ventral remnant in mm				Total surgery time in s				Time of using measuring tool in s	
	Mean	SD	Mean	SD	Mean	SD	Mean	SD	Mean	SD
Guidelines	with		without		with		without		with	
Experienced surgeon	1.34	1.47	1.41	0.98	254	79	250	126	33	12
Surgeon with limited experience	1.18	0.59	2.23	1.36	420	117	408	149	31	14

The mean total time for the positioning of the measuring tool and marking of the ventral canal floor with a needle was 33 ± 12 s for the experienced surgeon and 31 ± 14 s for the surgeon with limited experience (Table 2). There was no statistically significant difference between the experienced and the surgeon with limited experience (*p* = 0.698).

##### 3.2.2.2 Postoperative measures

The surgery was performed with similar precision for the experienced surgeon (*p* = 0.926), but the surgeon with limited experience achieved higher accuracy using the landmark values (*p* = 0.014). The mean difference to the VCF for the experienced surgeon was 1.41 ± 0.98 mm without landmark values and 1.34 ± 1.47 mm with landmark values. For the surgeon with limited experience, the mean difference was 2.23 ± 1.36 mm without landmark values and 1.18 ± 0.59 mm with landmark values (Table 2).

Both surgeons reported the landmark values were very helpful in 75% of the cases, little helpful in 20.8% of the cases, and not at all helpful in 4.2% of the cases overall, unrelated to the localization. The experienced surgeon found the landmark values helpful in 66.7% of the cases and little helpful in 33.3% of the cases. The surgeon with limited experience found the landmark values helpful in 83.4% of the cases, little helpful in 8.3% of the cases, and not at all helpful in 8.3% of the cases.

The spinal nerve roots stayed intact in 80% of the cases overall. The amount of intact and damaged spinal nerve roots was similar with and without landmark values for both surgeons.

## 4 Discussion

In this study, the objective was to identify landmark values aimed at enhancing precision in decompressive surgery of the thoracolumbar spine and to validate these findings through a cadaveric experiment. We successfully identified the accessory process as a reliable landmark that can be seamlessly translated from CT measurements to the surgical procedure. However, it is essential to note that these landmark values exhibit consistency only within the same breed. Specifically, the measurements focused on the distance between the DBAP and the VCF, resulting in a smaller ventral remnant without extending the duration of the surgical procedure. This, however, was only the case for the surgeon with limited experience. Consequently, we must reject the second hypothesis, which posited that an anatomical landmark would lead to decreased surgical time for both experienced and less experienced surgeons.

In this study, we assessed the DBAP-VCF value as an anatomical landmark to estimate the location of the VCF, aiming to enhance the precision of mini-hemilaminectomy procedures from the outset. We selected this measurement to gauge precision due to the predominant ventral or ventrolateral localization of extruded material (6, 7). The proximity to the ventral floor is expected to facilitate access to the ventral aspect of the vertebral canal and ease the decompression of the spinal cord, a critical consideration for mini-hemilaminectomy compared to traditional hemilaminectomy, given the smaller size of the access window. However, the impact of the presence and size of the ventral remnant on the risk of residual extruded disk material or increased iatrogenic damage due to limited access remains unclear. The authors note, that with an increasing step size, access and visualization of the entire ventral canal become more challenging without squeezing the spinal cord, but minimizing the step between the canal floor and mini-hemilaminectomy after creating the initial window may increase morbidity due to venous sinus bleeding and spinal nerve root damage (16, 17). The height of the vertebral sinus, approximately 0.45 mm from the VCF (14), suggests that a minimal remnant might even decrease the risk of bleeding. Therefore, precision is of paramount importance in the context of this study, especially given that the removal of residual bone fragments poses a potential risk of bleeding. The meticulous nature of the procedure is crucial to minimize any inadvertent damage and ensure optimal outcomes in decompressive surgery for intervertebral disk disease.

We used the measurement of the step from the mini-hemilaminectomy to the vertebral floor as a straightforward value for assessing precision. Despite using landmark values, we observed an overall remaining step of 1.26 mm, contrary to the expectation of complete elimination of any step. Several factors may contribute to this discrepancy, including the extrapolation of landmark values from CT measurements, variations in individual vertebral anatomy, and the 2D nature of the DBAP-VCF measurements not accounting for the three-dimensional shape of the vertebral arch and body. Additionally, the process of applying these measurements to the surgical field introduces a degree of imprecision.

We evaluated the postoperative integrity of the spinal nerve root. The spinal nerve roots of the corresponding vertebra exit the spinal canal in the cranial part of the vertebral foramen over the VCF just under the accessory process, which was used as a landmark in our study (18). The postoperative evaluation showed that 20% of the nerve roots were damaged, which is rather high compared to another study (19). One explanation could be that the procedures in our study were performed without magnification devices such as loupes or a surgical microscope, which can lead to more imprecision (20). In one case, the surgeon had the suspicion that there was preexisting damage to the nerve root secondary to maceration caused by the freezing and thawing of the cadaver and not to the surgery itself. Furthermore, the freezing and thawing procedure might have weakened the nerve roots and increased their tendency to rupture more easily. Additionally, the bone window in our study was performed using a pneumatic drill to access the inner cortex, but the canal itself was opened with a Kerrison rongeur. The use of Kerrison rongeur has been shown to lead to more spinal canal encroachment compared to the pneumatic drill (21), which is also a fact that we have to be aware of, especially in a clinical case where discus material is localized in the region of the nerve root. The effect of the usage of magnification devices and the pneumatic drill versus Kerrison rongeur for entering the spinal canal on the incidence of nerve root damage in a clinical patient was not part of the study and needs to be evaluated separately.

Surgeons with limited experience in the specific procedure face challenges such as restricted visibility of the surgical field, intricate anatomy, concerns about complications such as intraoperative sinus plexus bleeding, and the potential for iatrogenic spinal nerve root or spinal cord damage. These factors often contribute to prolonged surgery times, as surgeons with limited experience may proceed cautiously, palpating the VCF to estimate its location while advancing from the level of the accessory process. A study has indicated that residency-trained surgeons exhibit a learning curve, particularly in surgeries of increasing complexity (22). In this study, the surgeon with limited experience found the landmark values beneficial in over 80% of cases. Remarkably, the ventral border of the bone window was more accurately placed relative to the ventral canal floor compared to the experienced surgeon, suggesting that these landmark values, coupled with the measuring tool, can assist less experienced surgeons achieve better surgical precision. In an ideal case, this approach could aid in reducing intraoperative complications, surgical and anesthesia time, post-operative surgical site infection, and iatrogenic spinal cord damage (23).

While the hypothesis that the use of landmark values would lead to a shorter surgery time was rejected, the time to perform the osseous bone window itself was similar for both surgeons when using the measuring tool and landmark values. The mean time for measurements, although 32 s overall, did not significantly prolong the surgical time. This implies that the time spent on measurements outweighed the disadvantage in terms of surgical time, even though it did not decrease the overall duration. It is assumed that, even without using landmark values, correcting the window does not significantly extend the surgery time. However, potential implications for increased bleeding, not assessed in this study, should be considered. Application of this technique implies the necessity for patient-specific measurements pre-operatively.

Several limitations to this study should be acknowledged. The DBAP-VCF-value was evaluated using CT measurements from only 11 different chondrodystrophic breeds and a relatively small population size, precluding the immediate applicability of guidelines. Additionally, the DBAP-VCF-value was only evaluated for the vertebras from T11 to L4 and cannot be extrapolated for the vertebras from T1 to T10 and L5 to L7. The experimental procedure was conducted solely on cadavers, overlooking intraoperative factors such as hemorrhage, which may prolong surgery time and result from enlarging the window afterward. It is unclear how this might have affected the results of this study. The skin incision made in this experimental setup, spanning from T10 to L5, facilitated easier access to the spine compared to a clinical setting, potentially influencing the surgical procedure. Nonetheless, for testing multiple locations in one cadaver and given that soft tissues are typically not a major issue in this procedure, we deemed this approach appropriate.

In conclusion, this study identifies the DBAP-VCF as a valuable landmark for mini-hemilaminectomy, particularly aiding neurosurgeons with limited experience in increasing precision. However, the highly breed-specific nature of the landmark values necessitates individual evaluation for each breed.

## Data availability statement

The original contributions presented in the study are included in the article/supplementary material, further inquiries can be directed to the corresponding author.

## Ethics statement

Ethical approval was not required for the studies involving animals in accordance with the local legislation and institutional requirements because the study is a cadaveric study. Animals were euthanized unrelated to the study. Written informed consent was obtained from the owners for the participation of their animals in this study.

## Author contributions

SB: Conceptualization, Data curation, Formal Analysis, Investigation, Methodology, Project administration, Visualization, Writing – original draft. BH: Investigation, Writing – review & editing. SK: Conceptualization, Investigation, Methodology, Resources, Supervision, Validation, Writing – review & editing.
